# A Factor in Abdominal Disease

**Published:** 1920-03-20

**Authors:** 


### A FACTOR IN ABDOMINAL DISEASE.

### Theories and a Remedy.

In the current edition of the *British Journal of Surgery* Mr. G. E. Waugh presents a comparatively new conception of the origin of many abdominal diseases, and suggests a form of operative treatment which has apparently met with success in his hands. His views are attractive and demand consideration. They are essentially based on the theories of Sir Arbuthnot Lane, although the actual condition he describes has not hitherto been emphasised, and his treatment is certainly less drastic than colectomy.

### A Congenital Condition.

In many individuals (roughly 20 per cent.) the primitive mesentery of the ascending colon persists, owing to incomplete fusion of peritoneum in the course of development. The assumption of the upright position and associated influence of gravity tend to accentuate this state of affairs, and a pseudo-mesentery of fascia is superimposed on the congenital defect. This impairs the mechanical efficiency of the colon, and may adversely affect the function of other viscera with far-reaching results. In the ascending colon semi-solid material is driven vertically upwards, and, normally, the weight of this material is distributed by the firm fixation of the gut to the posterior abdominal wall. But, with the persistence of a mesentery, the gut must drag until this mesentery is taut, and the whole weight of the ascending colon and its heavy load is concentrated on the narrow linear attachment. The consequent disabilities are not likely to be serious during childhood, although the condition is primarily congenital; when the load increases with age, however, without a proportionate strengthening of its supporting mechanism, the defects are manifest.

### Mechanism of Strain.

The strain falls directly upon the anterior surface of the right kidney, the second part of the duodenum, and indirectly upon the pyloric region of the stomach, the cystic duct, and the gall bladder. The viscera affected may respond to the strain in one of two ways—they may be pulled out of their normal position, or their tissues may suffer. The former suggests tendency to gastroptosis or mobile kidney, the latter to gastric or duodenal ulcer. At operation, the association of a mobile ascending colon with mobility of the second part of the duodenum has been demonstrated, and the lines of strain radiating from the hepatic flexure to the gall bladder cystic duct and pylorus have been represented by puckering of the peritoneum on traction of the former.

Thus we are offered an explanation of the origin of many gastric, duodenal, biliary, and renal disabilities, and of the source of various forms of abdominal pain.

### Abdominal Pain.

As regards abdominal pain in general, the researches of Dr. A. F. Hurst have tended to show that, apart from inflammation of the peritoneum, visceral pain essentially originates in muscle. In other words, the sensation is due to a stretching or to an abnormal contraction of the muscle fibres of the viscera, rather than to any stimulation of the mucous membrane or the normal peritoneum. This view is not necessarily opposed to the "kink" theories. The negotiation of a kink by the contents of a hollow viscus necessitates an abnormal degree of muscular contraction in the same way as an intrinsic obstruction.

The operative technique adopted by Mr. Waugh amounts to a form of colopexy of the ascending colon. He makes an incision in the parietal peritoneum external to the mobile gut, and, preparing a bed on the posterior abdominal wall, fixes the colon thereto by suturing the new peritoneal edge to its anterior surface. This appears comparatively simple, and he, at any rate, has found its results satisfactory.

### Intestinal Stasis.

The whole conception of intestinal stasis and auto-intoxication has been brilliantly elaborated by Sir Arbuthnot Lane. The devastating effects are now too well recognised to need further emphasis. But his radical solution has only too frequently led in practice to the introduction of other unclean spirits, and the last state is worse than the first. We welcome, therefore, any reasonable attempt to solve the problem, notwithstanding a natural reservation of opinion.

With the close of the war period, in which the surgery of traumatic conditions came inevitably to occupy a prominent place among our surgical interests, the time has now arrived once more to concentrate upon that still unsolved problem, the origin of acute and chronic abdominal catastrophes. In 1914 the situation was, to say the least of it, vague in the extreme. Acceptance of the stasis ideas was on the wane, and although in the very process of partially disproving the Lane theories, further valuable facts have come to light, urgent requirements of hostilities have largely prevented advantage being taken of the bulk of information arising from this discussion.

### A Summary.

Therefore it may well serve some useful purpose to summarise the present ideas on abdominal surgery, and so, to a small extent, prepare the ground for a fresh attack upon these problems. Already we have drawn attention to the valuable conception of "a new abdominal disease," diverticulitis, which, if finally established, is an all-important addition. Now we have the contribution of Mr. Waugh, and in a future issue we hope to deal with those parts of the intestinal stasis theory which have withstood the test and criticisms of the past few years, and so provide an outline of the position as it stands to-day.

